# Analyzing homoeolog expression provides insights into the rediploidization event in gynogenetic hybrids of *Carassius auratus* red var. × *Cyprinus carpio*

**DOI:** 10.1038/s41598-017-14084-7

**Published:** 2017-10-20

**Authors:** Li Ren, Jialin Cui, Jing Wang, Hui Tan, Wuhui Li, Chenchen Tang, Qinbo Qin, Shaojun Liu

**Affiliations:** 1 State Key Laboratory of Developmental Biology of Freshwater Fish, Hunan Normal University, Changsha, 410081 Hunan China; 2 College of Life Sciences, Hunan Normal University, Changsha, 410081 Hunan China

## Abstract

Rediploidization is considered to be a part of the evolutionary history of allotetraploids, and resulted in the emergence of novel epigenetic regulatory activities. To study the changing patterns of gene expression following the reduction of a genome by 50%, we used RNA-seq and quantitative real-time PCR (qPCR) to investigate total gene expression and homoeolog expression in three hybrids of a *C*. *auratus* red var. (2n = 100, ♀) (R) and *C*. *carpio* (2n = 100, ♂) (C) (i.e., F_1_, F_18_, and G_4_) and their original parents. A comparison of homoeolog expression between G_4_ and F_18_ identified 7 genes (0.22%) that exhibited novel R/C homoeolog expression patterns in G_4_, while 4 genes (0.12%) were affected by R/C homoeolog silencing. We determined the direction and extent of the homoeolog expression bias (HEB). The C-HEB genes (i.e., *nrp1a* and *igf1)* and R-HEB genes (i.e., *fgf23* and *esm1*) provided insights into the effects of the dominance of one parental homoeolog expression on growth regulation. This dominance may contribute to the rapid growth of G_4_ fish. Our findings may be relevant for clarifying the relationship between growth heterosis and differences in homoeolog expression patterns.

## Introduction

All teleosts underwent at least three rounds of whole-genome duplication (WGD) (i.e., teleost-specific WGD) approximately 320 million years ago (Mya). For a few teleost species, including *Salmo salar* (approximately 80 Mya)^1^, *Cyprinus carpio* (approximately 8.2 Mya)^2^, and *Carassius auratus* red var. (approximately 18.49 Mya), a fourth WGD event occurred in a common ancestor^3^. Some studies have concluded that all WGD events were induced by hybridizations and led to polyploidizations, including emerge of allotetraploidizations origin from hybrid of *C*. *auratus* red var. × *C*. *carpio* ^4^, and autotetraploidizations which the genome duplication and the excretion of paternal chromosomes occurred in the F_1_ allotetraploid of *C*. *auratus* red var. × *Megalobrama amblycephala* ^1,2,5^. However, at least one previous study started to focus on subsequent genome size changes and diploidizations (i.e., returning to a diploid-like condition) in plants^6^. The silencing of homoeologs (i.e., originating from a parental gene), gene loss, and the formation of novel genes have contributed to the development of new specie^7^. These studies provided insights into the rediploidization process, which has not been fully characterized.

Rediploidization is considered the principal cause of rapid evolutionary reconciliations between two diverged genomes that accelerate the speciation process^1^. Some studies have revealed that the combining of two diverged genomes in hybrids can lead to the emergence of novel genotypes and phenotypes. In the latest relevant studies, analyses of total and homoeologous gene expression levels have been used to investigate phenotypic changes^8–10^. An analysis of the transcriptome shock efficiently revealed that considerable changes to gene regulatory networks occurred during a special stage. To fully characterize the rediploidization process and clarify the underlying molecular mechanism, the associated gene expression patterns will need to be investigated. These mechanisms may be related to allelic interactions and gene redundancy, and may involve non-coding RNA, DNA, and methylation and transcriptome changes, possibly leading to the appearance of novel traits^11–13^. For example, populations of gynogenetic diploid fish were observed to grow 30% faster than the parents of allotetraploid hybrid^14^. The analysis of homoeolog expression provides a useful platform to investigate phenotypic divergence related to growth and other traits after a rediploidization.

The hybridization between *C*. *auratus* red var. (2n = 100, ♀) (R) and *C*. *carpio* (2n = 100, ♂) (C) resulted in emergence of fertile allodiploid hybrids (F_1_ and F_2_, 2n = 100) and allotetraploid hybrids (F_3_-F_25_) because of the unreduced gametes from F_2_ hybrids^15^. Induction of gynogenesis in the fertile allotetraploid hybrids resulted in the emergence of fertile diploid gynogenetic fish lineage (G_1_-G_10_), in which the diploid gynogenetic fish produced diploid eggs with 100 chromosomes, and the fertilization of these eggs with UV-irradiated sperm from common carp resulted in the development of the subsequent diploid gynogenetic fish^16–18^. The diploid gynogenetic fish lineage (G_1_-G_10_) is the rediploidization event regarding the allotetraploid lineage. The study based on a fluorescence *in situ* hybridization (FISH) experiment revealed that half of the R and C (1:1) genomes were present in three hybrid lineages^17,19^. The allotetraploid hybrid lineage (from 2n to 4n) and the diploid gynogenetic lineage (back to 2n from 4n) with two genome level changes provided an excellent experimental system for the investigation of genetic process regarding the allotetraploidization and rediploidization events.

Studies of the three hybrid linages have focused on genetic mutations^19^, development of gonads and embryos^20,21^, and genotypes^19^, and confirmed the establishment of artificially cultivated and stable allopolyploid populations. The RNA-seq and quantitative real-time PCR (qPCR) techniques have gradually become more commonly used to study non-model organisms regarding the expression of homoeologs, which originate from different species^9,10,22,23^. In this study, the changes in homoeolog expression levels caused by a rediplodization event were investigated by comparison of homoeolog-specific single nucleotide polymorphisms (SNPs) between the reference genomes of *C*. *auratus* red var. and *C*. *carpio*. The direction and extent of the homoeolog expression bias (HEB) in diploid gynogenetic fish were then assessed by RNA-seq and qPCR. Additionally, we compared the diploid gynogenetic fish with allodiploid and allotetraploid hybrids of the *C*. *auratus* red var. × *C*. *carpio* to investigate the changes in homoeolog expression levels induced by different rediploidization mechanisms.

## Results

### Transcriptome sequencing

**Figure 1:**
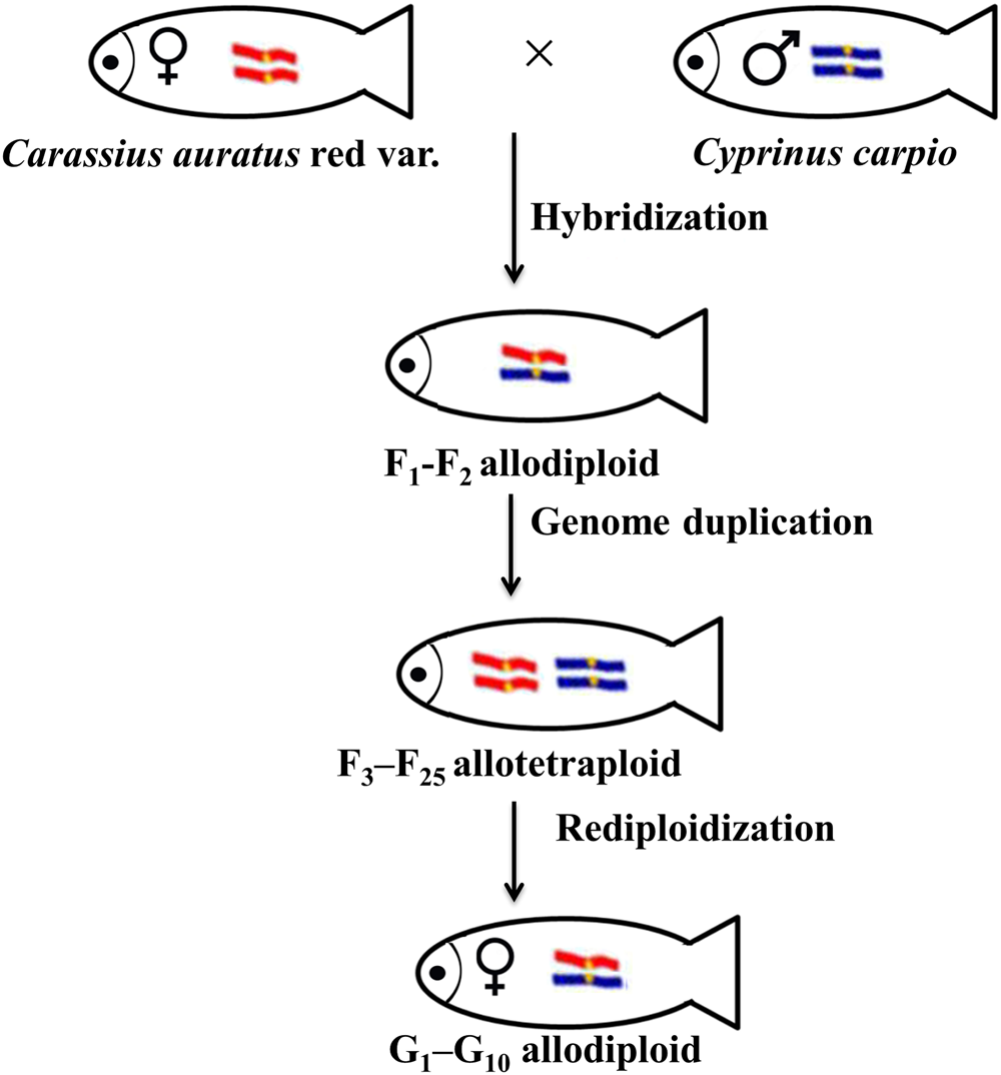
Genotypes of two cyprinid species and their three types of hybrid offspring, including allodiploids, allotetraploids and gynogenetic allodiploids, used for the comparison of homoeolog expression analysis.

To clarify the effects of rediploidization on transcript abundance, three hybrid lineages of the *C*. *auratus* red var. × *C*. *carpio* with different ploidy levels (i.e., F_1_, F_18_, and G_4_) and their original parents were analyzed^15,16^ (Table 1 and Fig. 1). Transcriptome sequencing produced 86.5 Gb of raw data for 15 libraries of the three hybrids and their original parents (Table S1). All short-read data were deposited in the Short Read Archive with the following accession numbers: SRX668436, SRX175397, SRX668453, SRX177691, SRX671568, SRX671569, SRX668467, SRX1610992, and SRX2347299.

**Table 1:** Genome and ploidy levels of two cyprinids and their three types of hybrids.

Groups	Genome	Ploidy level
*C*. *auratus* red var.	R_2_	diploid
*C*. *carpio*	C_2_	diploid
F_1_ hybrid	F_1_ (R × C)	diploid
F_18_ hybrid	F_18_ (R_2_ × C_2_)	tetraploid
Gynogenetic G_4_ hybrid	G (R × C)	diploid

### Statistical mapping of RNA-seq data

After eliminating the read adapters and low quality reads, clean reads (77.7%) from nine libraries for three hybrid lineages with different ploidy levels were mapped to reference transcriptomes of the maternal (R) and paternal (C) parents to obtain the total gene expression profiles. Clean reads of six *C*. *auratus* red var. and *C*. *carpio* libraries (84.4%) were mapped to their respective reference transcriptomes (Tables S2 and S3). The 11,998 genes commonly expressed among all samples were used for total gene expression analyses. Meanwhile, the homoeolog expression levels of the three hybrid lineages were detected using another method (described in method “Specific mapping of the R/C homoeologs”). The clean reads for the three hybrid lineages were mapped to reference transcriptomes of the parents based on the threshold values of the specific SNPs. The analysis of R and C homoeolog expression involved only 3,540 genes.

### Differential total gene expression in gynogenetic diploid fish

**Figure 2:**
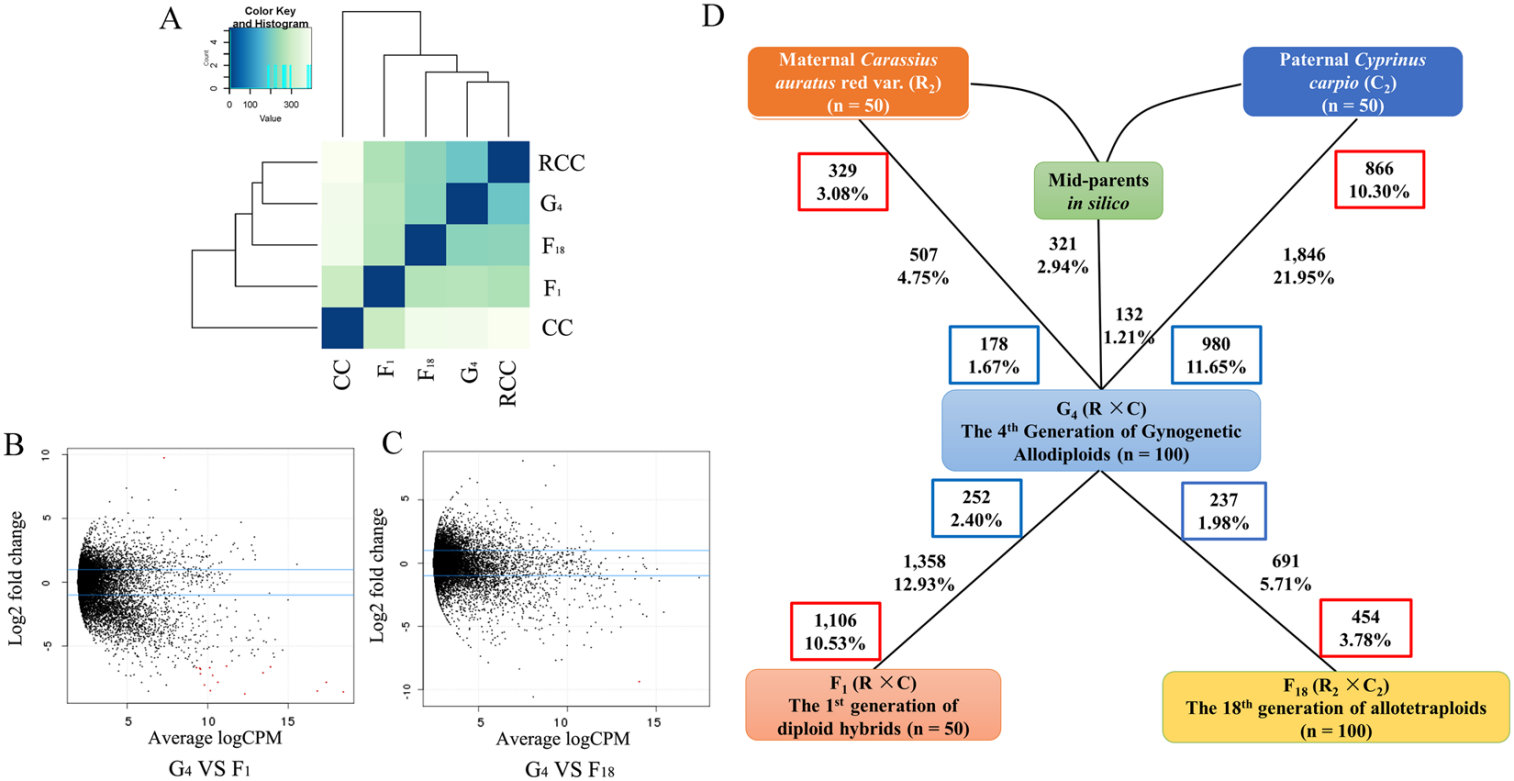
Global analysis of gene expression in two cyprinid species and their three types of hybrids. (**A**) Hierarchical clustering of gene expression in five groups revealed notable difference depending on the generation of hybrids and their ploidy levels which have considerable influence on global gene expression levels. Pearson correlation coefficients were calculated for all pairwise comparisons, and are presented in a heatmap following unsupervised clustering. (**B**) Comparison of the expression levels between G_4_ and F_1_ allodiploid hybrids. Black dots between two blue lines represent the genes with similar expression levels and those that are outside the blue lines represent the genes with significantly different expression levels (log_2_ FC > 2 and FDR < 0.05). (**C**) Comparison of the expression levels between G_4_ allodiploid and F_18_ allotetraploid hybrids. (**D**) Bold text indicates the total number and fraction of genes differentially expressed in each comparison. Proportions of the total number of differentially expressed genes that are up-regulated were also given in boxes. For example, 507 genes were differentially expressed between R and G_4_ groups. Of these, 329 were up-regulated in *C*. *auratus* red var. and 178 were up-regulated in G_4_.

A comparison of total gene expression among the three hybrid groups (i.e., F_1_, F_18_, and G_4_) based on hierarchical clustering revealed that the hybrid groups could clearly be separated from their parents (Fig. 2A). Additionally, the G_4_ allodiploid was highly correlated with its parental F_18_ allotetraploid (Fig. 2A). The MA-plots of the comparison of G_4_ with F_1_ and F_18_ are presented in Fig. 2B,C, respectively. Additionally, details regarding the differential total gene expression levels are provided in Fig. 2D. We identified 507 (4.75%) and 1,846 (21.95%) genes that were differentially expressed between G_4_ and the original maternal *C*. *auratus* red var. and paternal *C*. *carpio*, respectively. The analysis of total gene expression levels indicated that G_4_ was heavily biased toward the original maternal *C*. *auratus* red var.. Based on the comparison between G_4_ and F_18_, we identified 691 genes (5.71%) that were differentially expressed (Table 2), which was a consequence of rediploidization. In contrast, the comparison between G_4_ and F_1_ revealed more than double the number of differentially expressed genes (1,358, 12.93%) (Fig. 2D). Meanwhile, a comparison among G_4_, F_1_, and F_18_, and the original maternal *C*. *auratus* red var. revealed that the gene expression levels in G_4_ tended to be low (Fig. 2D). These results suggest that the global gene expression in G_4_ may have gradually decreased to a novel level among diploid lineages.

**Table 2:** Differences in total gene expression between the allotetraploid (F_18_) and gynogenetic allodiploid (G_4_) hybrids.

Comparison	Biological description	No. of genes	No. of growth genes
F_18 = _G_4_	No change	11,949 (78.0%)	142 (95.30%)
F_18_ > G_4_	Change due to rediploidization	454 (3.78%)	3 (2.01%)
F_18_ < G_4_	Change due to rediploidization	237 (1.98%)	4 (2.68%)
Total		11,998	149

We next investigated the expression level changes resulting from the rediploidization events and compared it with the hybridization and tetraploidization events (They had been described in latest reports^24^). Differences in total gene expression levels were identified by comparing each hybrid offspring with the original maternal *C*. *auratus* red var. and paternal *C*. *carpio*. Compared with the *C*. *carpio* expression levels, we detected 25 and 77 up- and down-regulated genes common to the three hybrid offspring (Fig. S1). However, only two down-regulated genes were identified in all three hybrid offspring lineages in a comparison with *C*. *auratus* red var. expression levels (Fig. S1).

### Novel expression pattern, silencing, and homoeolog expression bias

A gene ontology analysis (level 2) indicated that some genes were not simultaneously expressed in the liver of three hybrid individuals. These genes were mainly associated with metabolic or catalytic processes (Fig. S2). The novel expression pattern had been detected in 116 genes. Among of these, 83 genes (0.69% in G_4_) exhibited expression silencing in F_18_ and re-expressed in G_4_ (Table S4). While the remaining 33 genes (0.28%) expressed in F_18_, but silenced in G_4_ (Table S4). To investigate changes in homoeolog expression levels accompanying a rediploidization after an allotetraploidization, the R/C homoeolog expression patterns in F_1_, F_18_, and G_4_ were analyzed. During a comparison between G_4_ and F_18_, three (0.08%) and five (0.14%) genes were detected as R and C homoeologs with novel expression patterns in G_4_, respectively. We also identified two genes (0.06%) as silenced R homoeologs, and another two genes (0.06%) as silenced C homoeologs. These results suggest that a rediploidization accelerates the emergence of novel R/C homoeologous gene expression and silencing.

**Figure 3:**
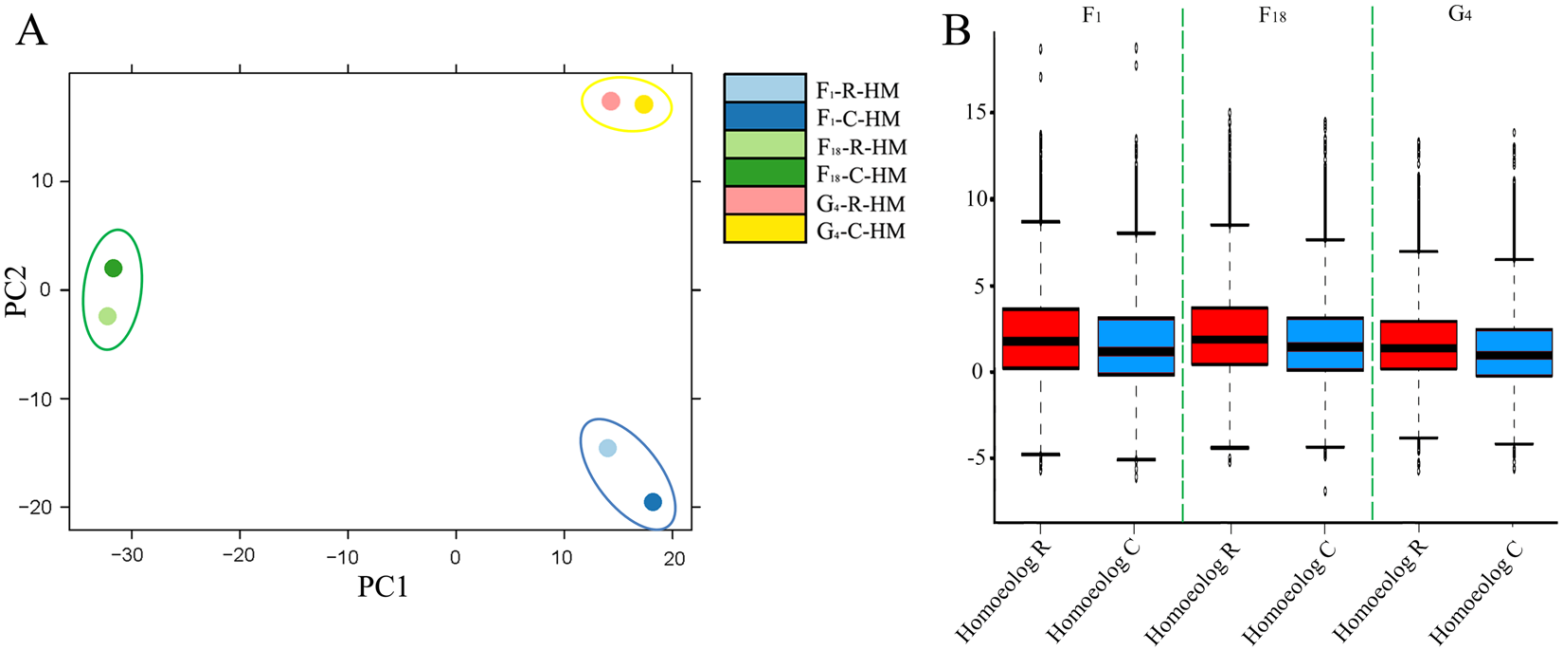
Distribution of the expression values of parental (R and C) homoeologs in their three types of hybrids. (**A**) Principal component analysis revealed that differences between the expression of maternal and paternal homoeologs were smaller in group G_4_ than that of in groups F_18_ and F_1_. F_1_ group had the most divergent expression of parental homoeologs. (**B**) Relative expression levels of parental homoeologs in their three types of hybrids.

**Figure 4:**
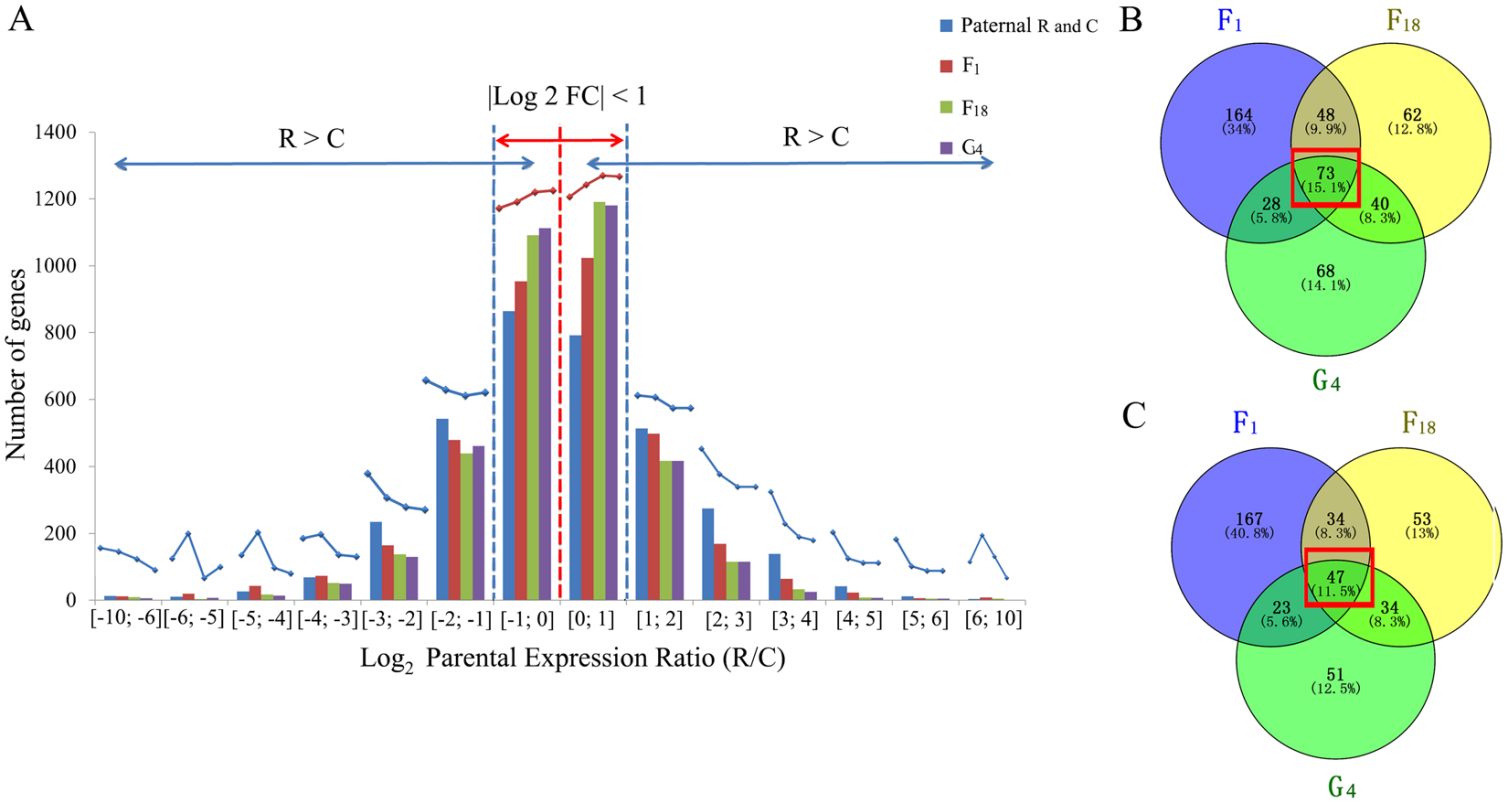
Distribution of the R/C-HEB and no-HEB genes in two cyprinid species and their three types of hybrids. (**A**) Comparison of the expression levels of parental (R and C) homoeologs in their three types of hybrids. The numbers of R-HEB and C-HEB genes changed with the changing ploidy levels of hybrids. The distribution of log_2_ FC values indicate that the expression levels of maternal and paternal homoeologs gradually approach to parental levels in hybrids during tetraploidy and rediploidization events. (**B**) Commonality of the R-HEB genes in the hybrids. (**C**) Commonality of the C-HEB genes in the hybrids.

Principal component analysis (PCA) confirmed a strong correlation between R and C homoeolog expression levels in all hybrids (Fig. 3A). Yet, the expression levels of R and C homoeologs in hybrids showed variation based on their genotype. The distribution of the R and C homoeolog expression levels for the three analyzed hybrids, which was consistent with the PCA results, is presented in Fig. 3B. The differences between the R and C homoeolog expression levels in G_4_ were smaller than those in F_18_. Additionally, the R and C homoeolog expression levels were more closely correlated in F_18_ than in F_1_. These results imply that the R and C homoeolog expression levels become increasingly related with the development of new generations with differing ploidy levels. After clarifying the R and C homoeolog expression levels, we obtained the distribution of the log_2_ fold change (FC) values between R and C in three hybrid lineages (Fig. 4A). After hybridizations, the phenomena of R- and C-HEB were observed for increasing numbers of genes as the number of generations increased. Meanwhile, the number of differentially expressed R and C homoeologs gradually decreased from F_1_ to G_4_ (Fig. 4A). For a more thorough comparison of R and C homoeolog expression levels, we divided the genes into those exhibiting HEB and those that did not (no-HEB) based on a specific differential gene expression threshold. After identifying the HEB genes in the three hybrids, we focused on R- and C-HEB genes to identify which genes maintained a strong HEB. According to the HEB analysis, 73 and 47 genes were determined to be R- and C-HEB genes in the three analyzed hybrid lineages, respectively (Fig. 4B,C).

To further investigate the effects of rediploidization on R and C homoeolog expression levels, we compared and classified only G_4_ and F_18_ genes. The HEB remained unchanged for most genes between F_18_ and G_4_. These genes were then divided into the following three categories: no-HEB (58.47%), R-HEB (13.84%), and C-HEB (4.21%) (Table 3). These observations suggest that the parental condition remained during a rediploidization. Meanwhile, the HEB of some genes changed because of a rediploidization. A total of 420 genes (11.86%) that exhibited HEB in F_18_, including 283 R-HEB genes (7.99%) and 137 C-HEB genes (3.87%), exhibited no-HEB in G_4_. Additionally, 260 (7.34%) and 143 (4.04%) genes with no-HEB in F_18_ exhibited R- and C-HEB in G_4_, respectively (Table 3). A few genes underwent considerable changes. For example, six genes (0.17%) changed from C-HEB to R-HEB, while the opposite change occurred in three genes (0.08%) (Table 3).

**Table 3:** Differences in homoeolog expression bias between the allotetraploid (F_18_) and gynogenetic allodiploid (G_4_) hybrids.

Comparison	Expression in F_18_	Expression in G_4_	No. of genes	No. of growth genes
F_18_ = G_4_	R = C (no-HEB)	R = C Parental condition	2,070 (58.47%)	20 (58.82%)
	R > C (R-HEB)	R > C Parental condition	490 (13.84%)	4 (11.76%)
	R < C (C-HEB)	R < C Parental condition	149 (4.21%)	0
F_18_ ≠ G_4_	R > C (R-HEB)	R = C No bias in progeny	283 (7.99%)	5 (14.71%)
	R < C (C-HEB)	R = C No bias in progeny	137 (3.87%)	2 (5.88%)
	R = C (no-HEB)	R > C Novel bias in progeny	260 (7.34%)	2 (5.88%)
	R = C (no-HEB)	R < C Novel bias in progeny	143 (4.04%)	1 (2.94%)
	R < C (C-HEB)	R > C Novel bias in progeny	6 (0.17%)	0
	R > C (R-HEB)	R < C Novel bias in progeny	3 (0.08%)	0
	Total number of genes		3,540	34
	Overall R-biased in progeny^a^		639 (18.52%)	5 (14.71%)
	Overall C-biased in progeny^a^		412 (11.94%)	2 (5.88%)
	Potential R-biased in progeny^b^		1629 (47.22%)	17 (50.00%)
	Potential C-biased in progeny^b^		860 (24.93%)	10 (29.41%)

R = C denotes equal expression; R > C and R < C denote R-biased and C-biased expression, respectively.

^a^Based on the significance differential homoeolog expression comparison of R and C homoeologues (*P* < 0.05 in comparisons; Fisher’s exact test).

^b^the ratio of R and C homoeologs greater than 1 was considered as potential R-biased in hybrids. Conversely, it represent as potential C-biased.

### Homoeolog expression bias of growth-regulating genes identified by RNA-seq and qPCR

A previous study concluded that the G_4_ fish grow 30% faster than the parental F_18_ fish^14^. Using RNA-seq technology, we analyzed the transcript levels of 92 growth-regulating genes (Table S5). The HEB status was obtained for only 34 of these genes (Table S6) because HEB was known for only 3,540 genes of the 11,998 genes included in the total gene expression analyses (Table 2). Most of the growth-regulating genes (142 genes, 95.30%) were not differentially expressed between G_4_ and F_18_. Only the dual specificity phosphatase 22 (*dusp22*) gene, growth hormone secretagogue receptor (*ghsra*) gene, fibroblast growth factor 19 (*fgf19*) gene, and glypican-4 (*gpc4*) gene were up-regulated in G_4_. In contrast, the bone morphogenetic protein 10 (*bmp10*) gene and two endothelial cell-specific molecule-1 (*esm1*) genes were down-regulated in G_4_ (Tables 2 and Table S5). Under the condition of no-HEB in paternal F_18_, the neuropilin 1 (*nrp1a*) gene exhibited C-HEB in G_4_, while the fibroblast growth factor 23 (*fgf23)* and *esm1* genes exhibited R-HEB (Tables 3 and Table S6).

**Figure 5:**
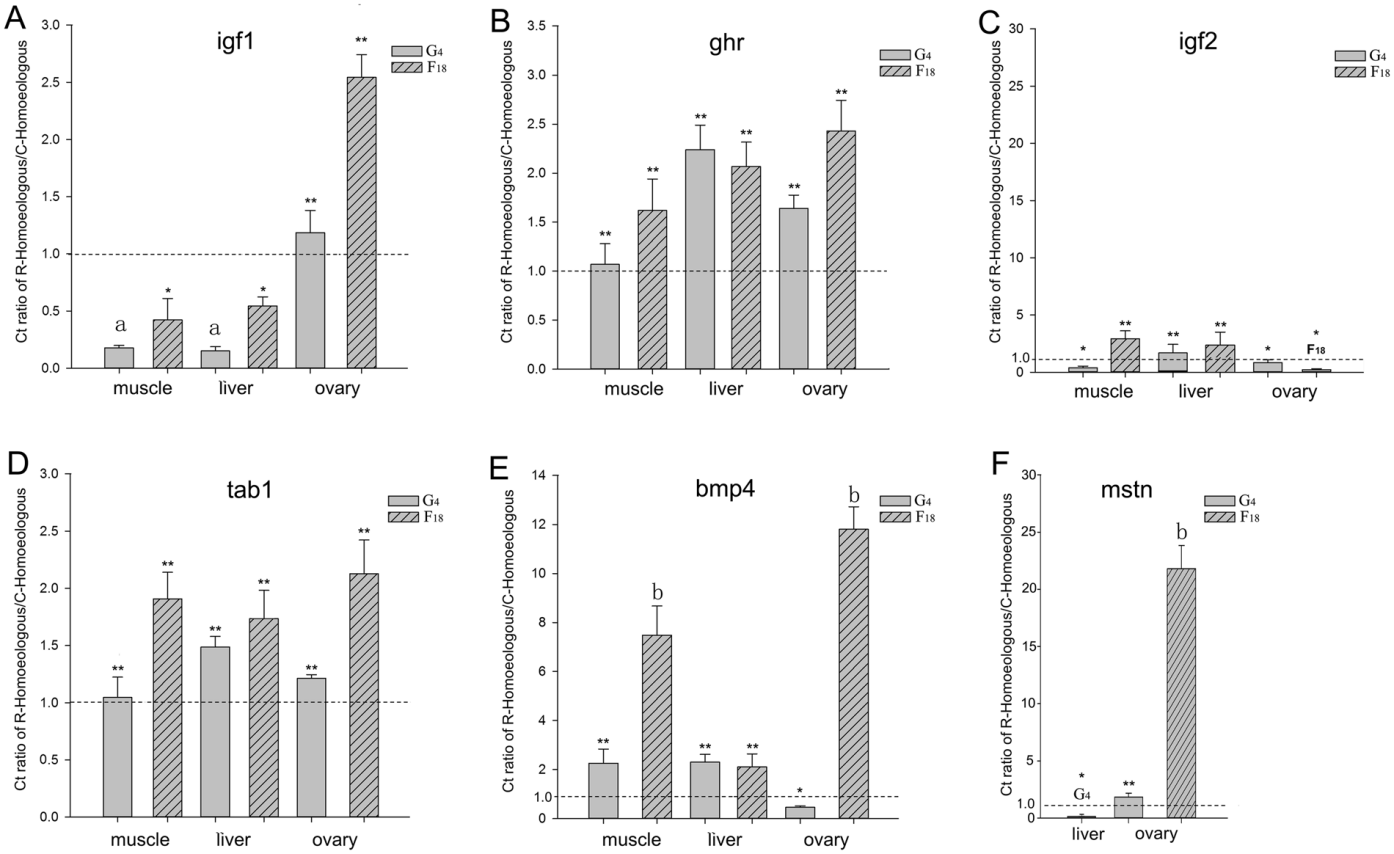
qPCR analysis of the six growth-regulating genes. The CT ratio of maternal (R) and paternal (C) homoeologs was based on tissue distribution analyses in G_4_ and F_18_ hybrid groups. (**A**) CT ratio of R homoeolog vs. C homoeolog for *igf1*. (**B**) CT ratio of R homoeolog vs. C homoeolog for *ghr*. (**C**) CT ratio of R homoeolog vs. C homoeolog for *igf2*. (**D**) CT ratio of R homoeolog vs. C homoeolog for *tab1*. (**E**) CT ratio of R homoeolog vs. C homoeolog for *bmp4*. (**F**) CT ratio of R homoeolog vs. C homoeolog for *mstn*. **potential R-HEB; *potential C-HEB. a: overall C-HEB; b: overall R-HEB. Comparative analysis revealed significant differences in gene expression (*P* < 0.05) (n = 3 for each group).

**Figure 6:**
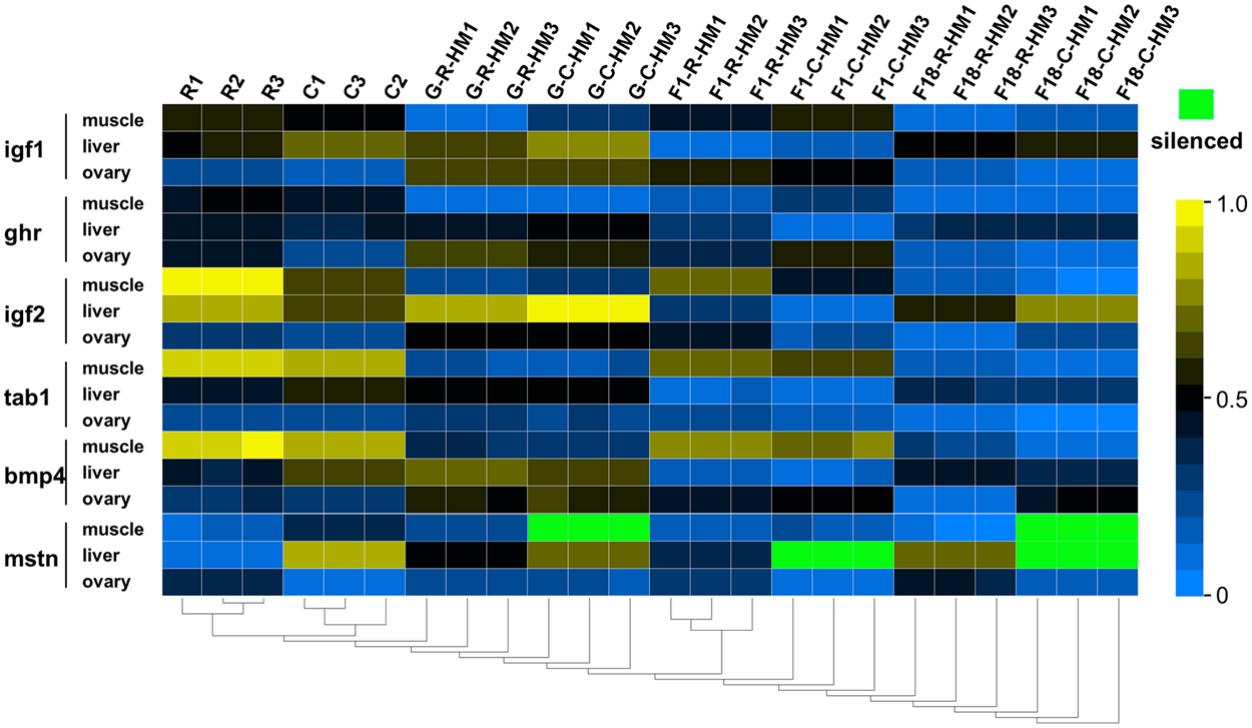
Hierarchical clustering analysis of homoeolog expression of six genes in various tissues of the parents (R 1–3 and C 1–3) and their three hybrid offspring (F_1_ 1–3, F_18_ 1–3, and G_4_ 1–3). Transcripts with high and low expression levels are indicated in yellow and blue, respectively.

To study the relationship between HEB and rapid growth, we determined the HEB of six growth regulated genes (i.e., *igf1*, *igf2*, *ghr*, *tab1*, *bmp4*, and *mstn*) in three tissues (i.e., liver, muscle, and ovaries) of G_4_ and F_18_ fish using homoeolog-specific qPCR (Fig. 5). Interestingly, we observed that the C homoeolog of the *mstn* gene was silenced in the muscle of G_4_ and F_18_ fish (Fig. 6). Novel expression patterns of the C homoeolog of the *mstn* gene were observed in the liver of G_4_ fish, while this homoeolog was silenced in F_18_ (Fig. 6). We also observed differences in the extent of R/C-HEB in the three analyzed tissues (Fig. 6). Specifically, *igf1* exhibited an overall R-HEB in the muscle and liver of G_4_ fish, while *bmp4* and *mstn* exhibited an overall C-HEB in the ovaries of F_18_ fish. In summary, R-HEB was the predominant expression bias in the liver, muscle, and ovaries.

## Discussion

### Continuous changes to homoeolog expression accompany to new generations with different ploidy levels

In this study, the initial hybridization involved the merging of *C*. *carpio* and *C*. *auratus* red var. genomes. Following a genome duplication in the F_2_ diploid hybrid gametes, a bisexual fertile allotetraploid population was obtained^15^. Gynogenesis was then exploited to breed the G_1_ diploid hybrid, in which the genome consisted of only half of the allotetraploid genome^16^. The three hybrids and the two associated genome-level changes enabled us to elucidate the relationship between genomic variation and growth differences in hybrids.

Characterizing the structural and functional changes in hybrids with different ploidy levels is a major challenge because of the complexities of genome structures and regulatory pathways. In particular, it is still unclear whether the two original parental genomes contribute equally to the gene expression levels of hybrids. We explored this uncertainty in detail using a systematic approach for dissecting the relative contributions of two homoeologs (i.e., R and C) to total gene expression levels. We also investigated the changes to homoeolog expression accompanying a genome duplication and rediploidization events. Only a few studies have focused on the differential expression of homoeologs in vertebrates because of a lack of fertilized hybrids. However, studying homoeologs originating from different species has been considered useful for clarifying the genetic structure and specific phenotypic changes occurring in hybrids with differing ploidy levels^25,26^. Subsequent genome-wide assessments have relied primarily on microarrays, which may not be appropriate for distinguishing the expression of closely related genes^10,27^. Therefore, these studies have been unable to separate the contributions of different homoeologs, particularly the R and C homoeologs originating from two diploid parental species. In this study, we used two novel analyses based on RNA-seq and qPCR to study the HEB under the condition that the gene transcripts from the parental species could be distinguished. A subsequent analysis of growth-regulating genes provided insights into growth heterosis resulting from a rediploidization.

The genome reduction following a hybridization altered the parental allotetraploid gene expression levels. Our total gene expression analysis revealed that 5.97% of the genes were differentially expressed between G_4_ and F_18_, while 21.5% of the genes were differentially expressed between F_1_ and F_18_ (Fig. 2). Additionally, analyses of homoeolog expression indicated that the number of genes exhibiting HEB decreased in new generations with ploidy-level changes (Fig. 2). These observations imply that a rediploidization leads to novel expression patterns, which are not simply the result of a reversal of the tetraploidization process. Compared with their parents, a new population was observed to exhibit differences related to growth^14^, random amplification of polymorphic DNA and microsatellite characteristics^14^, and reproduction^17^. These changes were closely related to gene expression, especially homoeolog expression levels. Additionally, the maternal R-HEB phenomenon was detected in three hybrids (Table 3)^24^. Maternal expression bias is frequently observed in hybrid fish, including the *M*. *amblycephala* × *Culter alburnus* ^8^, *Oncorhynchus mykiss* ^28^, and *S*. *salar* ^28^ hybrids. Our PCA results indicated that the R and C homoeolog expression levels were increasingly closely linked during the reconstruction of the hybrid genotype and the development of new generations (Fig. 3). The hybrid genome gradually started to change, with the gene expression levels inherited from the original parents slowly reaching a similar level. These gene expression level changes suggest the hybrid lineages tended to reach a steady state. However, tetraploidizations and rediploidizations were associated with increases in the number of R-HEB genes. This change also influenced hybrid phenotypes, and induced various genetic changes conducive for adaptations. Recent studies have deduced the possible mechanisms underlying changes to total gene and homoeolog expression levels. Allelic interactions and gene redundancies were considered the major causes of alterations to non-coding RNA, DNA, and methylation, which resulted in additional changes to the hybrid transcriptomes^11,29^.

An analysis of homoeolog expression related to hybrid characteristics has produced relatively precise information regarding the associated regulatory mechanisms^30^. To investigate the transcriptome shock caused by a rediploidization and the resulting increase in the growth rate of G_4_ fish, we analyzed the expression of growth-regulating genes. The four up-regulated and three down-regulated genes enabled additional investigations into the relationship between gene expression levels and growth rates. However, we did not conduct any related phenotypic analyses. Thus, we were only able to obtain the annotation details of these genes (i.e., *dusp22*, *ghsra*, *fgf19*, *esm1*, and *gpc4*), which are important for controlling cell division and regulating growth^31–34^. Furthermore, *bmp10* encodes a potent inhibitor of endothelial cell migration and growth^35^. The down-regulated expression of this gene likely promotes growth (Tables 2 and Table S5). We used the analysis of HEB among growth-regulating genes to investigate the relationship between HEB and phenotypes, and to characterize the mechanism underlying heterosis in polyploid hybrids (Tables 3 and Table S6). Our RNA-seq results suggested that *nrp1a* was associated with C-HEB, while *fgf23* and *esm1* were related to R-HEB (Tables 3 and Table S6). An assessment of the localization of the expression of six key growth-regulating genes revealed that *igf1* exhibits C-HEB in the muscle and liver. The original paternal parent *C*. *carpio* grows faster than the maternal parent *C*. *auratus* red var.^24^. The C-HEB of *igf1* likely promotes the dominance of the paternal growth characteristic in G_4_, possibly inducing a rapid growth rate.

Interestingly, the diploid G_4_ retained the inherited characteristics of the parental F_18_. The silencing of the C homoeolog was also observed for the *mstn* gene (Figs 5 and 6). This may have been due to genomic imprinting, implying that the regulation of gene expression is mediated by one parental genome, while the genetic material inherited from the other parent is silenced in the hybrid^36^. Some genes in hybrids always exhibit single-genome–mediated expression^36^. An earlier study concluded that mutations in *mstn* always result in increased muscle mass and strength in vertebrates, making these individuals considerably stronger than their peers^37^. However, we determined that the expression pattern for a C homoeolog of *mstn* in the liver of G_4_ fish differed from that of F_18_ fish. The re-expression of *mstn* reflected the new regulatory mechanism influencing growth that may contribute to an increased growth rate in G_4_ individuals. This *mstn* expression pattern was similar to that observed in F_1_^24^. However, additional studies are required to verify that a rediploidization accelerates the emergence of new phenotypes *via* changes to the HEB and homoeolog silencing.

## Conclusion

Gynogenetic diploid offspring provide unique opportunities to study the evolutionary effects of rediploidization. The associated transcriptome shock was clarified by a novel analysis of genes exhibiting HEB. In this study, we examined this phenomenon in detail using qPCR and homoeolog-specific sequences in the transcriptome. Our data revealed the direction and extent of HEBs and homoeolog silencing changes accompanying to hybridization, tetraploidization, and rediploidization. Additionally, our findings may be useful for more comprehensively characterizing of the relationship between novel growth phenotypes and homoeolog expression differences. Additional studies on this topic might contribute to elucidation of the mechanism regulating growth heterosis.

## Materials and Methods

### Animal materials

Fertile allodiploid hybrids (F_1_ and F_2_, 2n = 100) were obtained from the hybridization between *C*. *auratus* red var. (2n = 100, ♀) (R) and *C*. *carpio* (2n = 100, ♂). Then, the unreduced gametes from F_2_ hybrids lead to emergence of bisexual fertile allotetraploid hybrids (4n = 200). Until now, the allotetraploid lineage continued into F_25_ by successive self-crossing. A fertile diploid gynogenetic fish lineage was obtained from the induction of gynogenesis in the female fertile allotetraploid hybrids (2n = 100). The fertilization of gynogenetic fish was performed with the eggs with UV-irradiated sperm from common carp. This technology had ensured the continuous of diploid gynogenetic fish lineage (G_1_-G_10_), which could be considered as the model of rediploidization event regarding the allotetraploid lineage. The results of FISH showed that the genotype of three hybrid lineages were half of the R and C (1:1) genomes (Table 1).

All experiments (2012–2015) were approved by the Animal Care Committee of Hunan Normal University. We followed the animal experimentation guidelines of the Science and Technology Bureau of China. The experimental fish were kept in an indoor freshwater tank which maintained to carry suitable environmental conditions in terms of photo-period, water temperatures, forage etc. in the Engineering Center of Polyploidy Fish Breeding of the National Education Ministry located at Hunan Normal University, China. Fish were deeply anesthetized with 100 mg/L MS-222 (Sigma-Aldrich, St. Louis, Missouri, USA) before being dissected. Three mature females from each ploidy group, including diploid *C*. *auratus* red var., diploid *C*. *carpio*, F_1_ allodiploid hybrids of *C*. *auratus* red var. × *C*. *carpio*, F_18_ allotetraploid hybrids of *C*. *auratus* red var. × *C*. *carpio*, and G_4_ allodiploid hybrids of *C*. *auratus* red var. × *C*. *carpio* (2-year-old individuals) were collected.

Their ploidy levels were confirmed by measuring the DNA contents of their erythrocytes via flow cytometry and by direct counting of chromosome numbers from metaphase spreads. To prepare metaphase spreads, we cultured the red blood in DMEM solution for 68–72 h at 25.5 °C and 5% CO_2_. Cells were harvested by centrifugation, and then treated in a hypotonic solution (0.075 M KCl) at 26 °C for 25–30 min. Samples were fixed in a methanol–acetic acid (3:1, v/v) solution with three changes. Then, the samples were placed on cold slides, air-dried, and stained in 4% Giemsa solution for 30 min. The chromosome numbers were observed in metaphase spreads of 15 individuals to determine the ploidy levels.

### cDNA generation, library construction, and RNA sequencing

After anesthetizing the fish with 2-phenoxyethanol, the liver, muscle, and ovary tissues were excised and immediately placed in RNAlater for storage based on the manufacturer’s instructions (Ambion Life Technologies, Carlsbad, CA, USA). Total RNA extracts were treated from the harvested tissues according to a standard Trizol protocol (Invitrogen) after the RNAlater was removed. Total RNA was treated with a DNA-free™ DNA Removal Kit (Ambion) to remove any contaminating genomic DNA. The purified RNA was quantified using a 2100 Bioanalyzer system (Agilent, Santa Clara, CA, USA).

We fragmented 2 μg isolated mRNA with fragmentation buffer. The resulting short fragments were reverse transcribed and amplified to produce cDNA. An Illumina RNA-seq library was prepared according to a standard high-throughput method^38^. The cDNA library concentration and quality were assessed by Qbit (Invitrogen) and the Agilent 2100 Bioanalyzer, after which the library was sequenced using the Illumina HiSeq. 2000 platform. The RNA-seq experiment was conducted with three biological replicates. The transcriptome data were generated using a 101-bp paired-end setting. After removing the read adapters and low quality reads, clean reads from each library were examined by using the FastQC program (Version 0.11.3)^39^. Afterwards, principal component analysis (PCA) was performed on 15 liver transcriptomes to elucidate the contribution of each transcript to different classes in the 15 liver transcriptomes^40^.

### Specific mapping of the R/C homoeologs

For detection of changes in homoeolog expression due to a rediploidization event, we obtained the genome of the maternal parent *C*. *auratus* red var. (http://rd.biocloud.org.cn/) (39,069 transcripts) and the genome of the paternal parent *C*. *carpio* (http://www.carpbase.org/) (52,610 transcripts). A database of full-length transcripts was used as a resource for gene expression details (Table S2). Additionally, analyses of putative orthologs between R and C may help to identify true orthologs. Thus, the R and C sequences were aligned by using the reciprocal BLAST (BLASTN) hit method, with an e-value cut off of 1e^−20^ ^41^. Two sequences were defined as orthologs if each one was the best hit for the other, and the aligned sequences contained at least 300 bp. We used putative R and C orthologs as the reference sequences for detecting the homoeolog expression of hybrids. By using custom perl scripts, we calculated the homoeolog expression levels over the SNPs between the R and C orthologs were applied for calculating homoeolog expression levels. The mapping of reads to the reference transcriptome for three hybrids and their original parents was completed by means of TopHat2 program^42^.

### Analyses of homoeolog expression bias

Prior to analyses, the expression level data were normalized by using Cufflink program (version 2.1.0)^43^. We also restricted the data analysis with the number of read counts (≥ 1) of genes to remove the negative effects of background expression noise in all biological replicates. The abundance or the coverage of each transcript was normalized by using the number of reads per kilobase of exon per million mapped reads (RPKM)^44^. Gene expression levels were estimated based on the RPKM values of the reads. The false discovery rate (FDR) was used for determining the threshold P value in multiple tests and analyses. Meanwhile, unigenes with an FDR ≤ 0.05 and a fold change > 2 were considered differentially expressed genes. The mapping results were analyzed with the DEGseq package of the R program (version 2.13) (R Foundation for Statistical Computing, Vienna, Austria)^45^.

Comparisons of the R/C homoeolog expression levels among three hybrids were used to identify the genes with potentially novel expression patterns (e.g., new expression of a gene in the liver) and silenced genes in the hybrids according to the standards described by Yoo *et al*.^9^. To screen the newly expressed or silenced genes, we analyzed the homoeolog expression categories associated with these genes, which were simultaneously expressed among the three hybrids and their original parents. For homoeolog expression bias (HEB) analyses, we compared R and C homoeolog expression levels in the three hybrids. The log_2_ FC of homoeologue expression value (R vs C) > 2 had been considered as the threshold values of R-HEB. Contrarily, the log_2_ FC (C vs R) > 2 had been considered as the threshold values of C-HEB. The extent and direction of differential homoeolog expression were assessed according to *Ren et al*.^24^.

### Homoeolog-specific qPCR

To elucidate the relationship between HEB and rapid growth, we examined the HEB of six important genes (i.e., *igf1*, *igf2*, *ghr*, *tab1*, *bmp4*, and *mstn*) previously observed to regulate growth rates^13,46,47^. However, HEB information was lacking for some of these genes. So the homoeolog-specific qPCR had been used to detect the homoeolog expression level. Total RNA was extracted from the liver, muscle, and ovaries. First-strand cDNA was synthesized using AMV reverse transcriptase (Fermentas, Canada) with an oligo (dT)_12–18_ primer at 42 °C for 60 min and 70 °C for 5 min. To further detect the changes in homoeolog expression pattern of growth-regulating genes, we obtained sequence information for the R and C transcripts of a housekeeping gene (i.e., *β-actin*) and six key growth-regulating genes (i.e., *igf1*, *igf2*, *ghr*, *tab1*, *bmp4*, and *mstn*) from the *C*. *auratus* red var. and *C*. *carpio* transcripts. A comparison of the R and C homoeolog sequences by using Bioedit program (version 7.0.9)^48^ revealed the presence of homoeolog-specific loci in all seven genes. The regions containing homoeolog-specific loci were used to design R/C homoeolog-specific primers for qPCR analysis^24^. To obtain highly sensitive specific primers, we completed cross amplifications by using ABI Prism 7500 Sequence Detection System (Applied Biosystems, USA)^24^. The PCR amplification conditions were as follows: 50 °C for 5 min, 95 °C for 10 min, and 40 cycles at 95 °C for 15 s and 60 °C for 45 s. Each test was conducted three times to improve accuracy of the results. Finally, relative quantities were calculated, and a melting curve analysis was used to verify the generation of a single product. Each sample was used in triplicate for the assays and to generate standard curves. The expression data for each homoeologous gene was normalized against that of *β-actin* according to 2^−ΔΔCt^ method^49^. The *β-actin* expression level in the hybrids was estimated by using the ratio of transcript abundance to gene copy number using PCR and qPCR conducted with co-extracted DNA and RNA samples. The *β-actin* expression levels in somatic organs and gonads were similar among the three hybrids and their original parents^50,51^.

### Data deposition

The transcriptome data were submitted to NCBI (accession number: accession numbers: SRX668436, SRX175397, SRX668453, SRX177691, SRX671568, SRX671569, SRX668467, SRX1610992, and SRX2347299). The remaining data are available within the article and its Supplementary Information files or available from the authors upon request.

## Electronic supplementary material

Supplementary tables

Supplementary Information
