# Supplementary material for: Analyzing homoeolog expression provides insights into the rediploidization event in gynogenetic hybrids of Carassius auratus red var. × Cyprinus carpio
Source: Sci Rep. 2017 Oct 20;7:13679. doi: 10.1038/s41598-017-14084-7 (PMC5651915; doi:10.1038/s41598-017-14084-7)
Supplement: Supplementary file 2 — Supplementary Information [file 41598_2017_14084_MOESM2_ESM.doc]

**Analyzing homoeolog expression provides insights into the rediploidization event in gynogenetic hybrids of *Carassius auratus* red var. × *Cyprinus* *carpio***

**Li Ren^1,2*^, Jialin Cui^1,2*^, Jing Wang^1,2^, Hui Tan^1,2^, Wuhui Li^1,2^, Chenchen Tang^1,2^, Qinbo Qin^1,2^ and Shaojun Liu^1,2§^**

^1^State Key Laboratory of Developmental Biology of Freshwater Fish, Hunan Normal University, Changsha 410081, Hunan, P.R. China.

^2^College of Life Sciences, Hunan Normal University, Changsha, 410081, Hunan, P.R. China.

**^§^**Corresponding author:

Professor Shaojun Liu: lsj@hunnu.edu.cn

State Key Laboratory of Developmental Biology of Freshwater Fish, Hunan Normal University

Changsha 410081, China

Tel/Fax: +86-073188873074

^*^These authors contributed equally to this work.

**Supplementary table 1:** Summary statistics of the transcriptome data in liver

|  | Number of libraries | Total number of reads | Total number of bases (Gb) | Read length | Data accession numbers |
| --- | --- | --- | --- | --- | --- |
| *C. auratus* red var. (♀) | Illumina × 3 | 1.92×10^8^ | 17.3 | 101*2, 87*2 | SRX668453, SRX177691 |
| *C. carpio* (♂) | Illumina × 3 | 1.93×10^8^ | 18.5 | 101*2, 87*2 | SRX668436, SRX175397 |
| F_1_ allodiploid | Illumina × 3 | 2.07×10^8^ | 19.9 | 101*2, 87*2 | SRX671568, SRX671569 |
| F_18_ allotetraploid | Illumina × 3 | 1.77×10^8^ | 17.0 | 101*2, 87*2 | SRX668467, SRX1610992 |
| G_4_ allodiploid | Illumina × 3 | 1.36×10^8^ | 13.8 | 101*2 | SRX2347299 |

**Supplementary table 2.** The basic information of maternal *Carassius auratus* red var. and paternal *Cyprinus carpio* reference coding sequences in our study

|  | *C. auratus* red var. | *C. carpio* |
| --- | --- | --- |
| Number of annotated genes | 20,169 | 20,235 |
| Number of contig | 39,069 | 52,610 |
| Large contig (≥ 1000bp) | 23,226 | 27,751 |
| Max contig length (bp) | 90,843 | 760,200 |
| Mean contig length (bp) | 1,637.7 | 1,507.6 |
| N50 length (bp) | 2,259 | 2,164 |

**Supplementary table 3**. The summary of mapping to reference transcripts

| Samples | Total Reads | Mapped Reads (Million) | The ratio of Mapped Reads |
| --- | --- | --- | --- |
| *C. auratus* red var. | 1.92×10^8^ | 1.62×10^8^ | 84.45% |
| *C. carpio* | 1.93×10^8^ | 1.63×10^8^ | 84.54% |
| F_1_ allodiploid | 2.07×10^8^ | 1.67×10^8^ | 80.73% |
| F_18_ allotetraploid | 1.77×10^8^ | 1.37×10^8^ | 77.46% |
| G_4_ allodiploid | 1.36×10^8^ | 1.02×10^8^ | 75.07% |

**Supplementary table 4**. The number of total expression genes showing the patterns of novel expression and expression silencing between G_4_ with their parental F_18_ (at threshold of 10 reads homoeolog per million reads)

| Taxa | Novel expression (%) | Novel expression in growth genes (%) | Silencing (%) | Silencing in growth genes (%) |
| --- | --- | --- | --- | --- |
| G_4_ | 83 (0.69%) | 0 | 33 (0.28%) | 1 (0.01%) |

**Supplementary Figure**


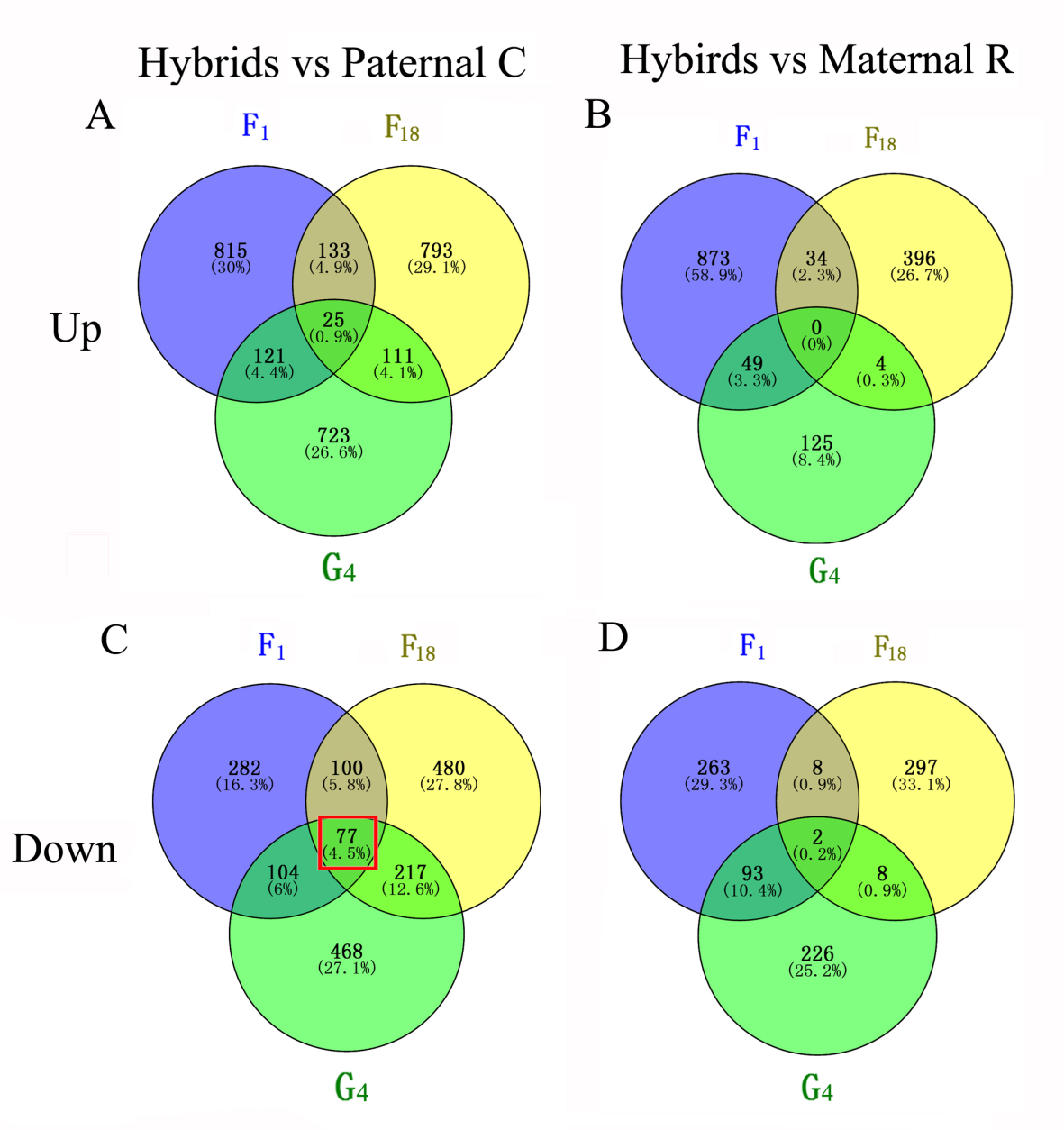


Supplementary Fig. 1. Differential global gene expression in comparison of three hybrids and their original parents. A. the share up-regulated genes in comparison of three hybrids and original paternal *C. carpio*. B. the share up-regulated genes in comparison of three hybrids and original maternal *C. auratus* red var.. C. the share down-regulated genes in comparison of three hybrids and original paternal *C. carpio*. D. the share down-regulated genes in comparison of three hybrids and original maternal *C. auratus* red var..


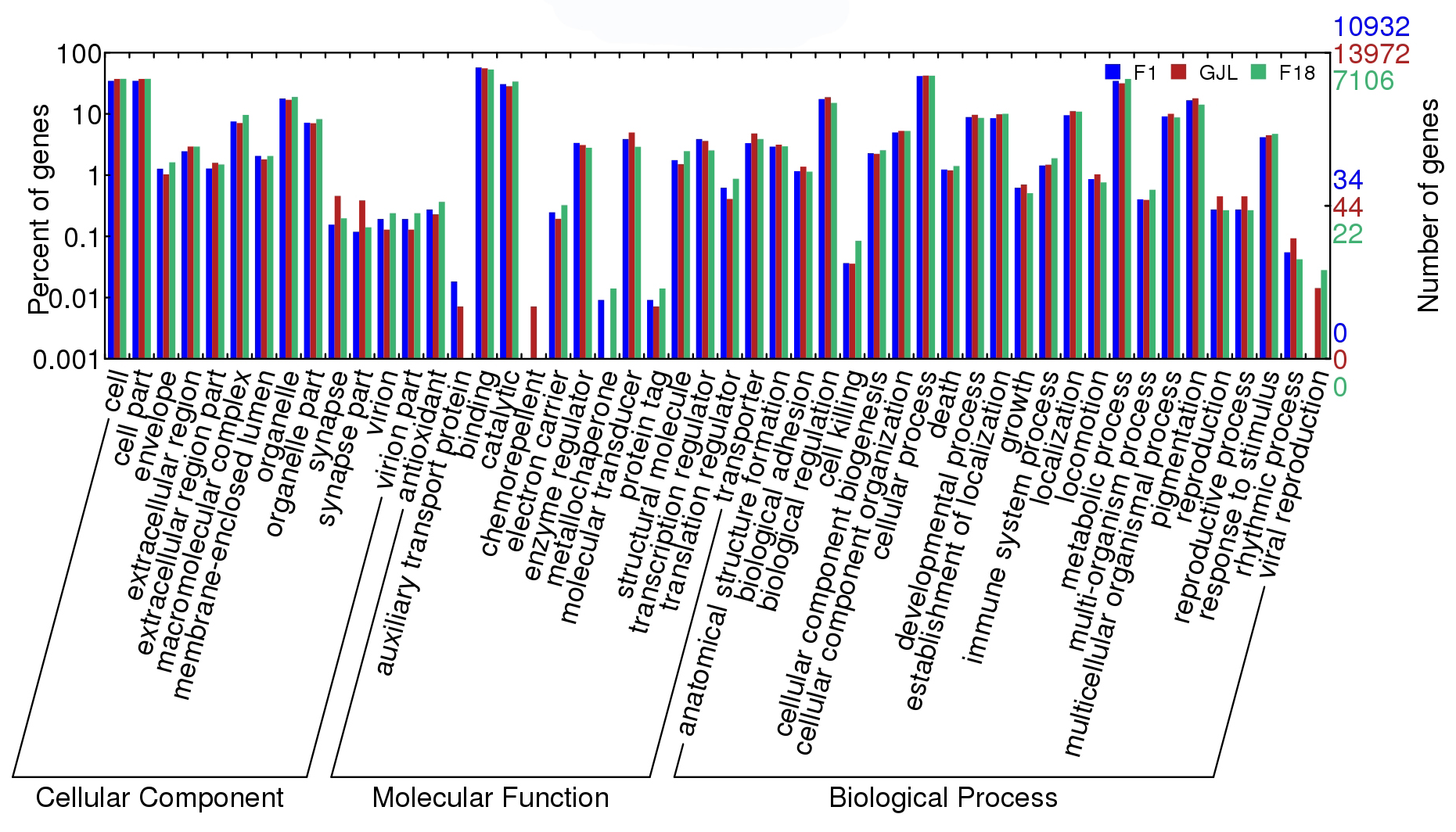


Supplementary Fig. 2. Gene ontology (GO) (level 2) assignments for expression genes in three hybrids with different ploidy level.
